# Effects of phosphoglycerate kinase 1 and pyruvate kinase M2 on metabolism and physiochemical changes in postmortem muscle

**DOI:** 10.1016/j.fochx.2024.101125

**Published:** 2024-01-09

**Authors:** Caiyan Huang, Dequan Zhang, Christophe Blecker, Yingxin Zhao, Can Xiang, Zhenyu Wang, Shaobo Li, Li Chen

**Affiliations:** a Institute of Food Science and Technology, Chinese Academy of Agriculture Sciences, Key Laboratory of Agro-Products Quality & Safety Harvest, Storage, Transportation, Management and Control, Ministry of Agriculture and Rural Affairs, Beijing 100193, China; b Gembloux Agro-Bio Tech, Unit of Food Science and Formulation, University of Liège, Avenue de la Faculté d’Agronomie 2, Gembloux B-5030, Belgium; c Department of Agricultural, Food and Nutritional Science, University of Alberta, Edmonton, Alberta T6G 2P5, Canada; d Institute of Food Science and Biotechnology, Department of Flavor Chemistry, University of Hohenheim, Fruwirthstraße 12, 70599 Stuttgart, Germany

**Keywords:** PGK1, PKM2, Inhibitors, Activators, Postmortem muscle, Glycolysis

## Abstract

The objective of this work was to investigate the influence of phosphoglycerate kinase-1 (PGK1) and pyruvate kinase-M2 (PKM2) activity on glycolysis, myofibrillar proteins, calpain system, and apoptosis pathways of postmortem muscle. The activity of PGK1 and PKM2 was regulated by their inhibitors and activators to construct the postmortem glycolysis *vitro* model and then incubated at 4 °C for 24 h. The results showed that compared to PGK1 and PKM2 inhibitors groups, the addition of PGK1 and PKM2 activators could accelerate glycogen consumption, ATP and lactate production, while declining pH value. Moreover, the addition of PGK1 and PKM2 activators could increase desmin degradation, μ-calpain activity, and caspase-3 abundance. Interestingly, troponin-T degradation was significantly increased both in PKM2 inhibitor and activator groups. It was suggested that PGK1 and PKM2 might be used as robust indicators to regulate meat quality by affecting the glycolysis, myofibrillar proteins, μ-calpain and apoptosis pathways in postmortem muscle.

## Highlights

• Glycolysis was promoted in PGK1 and PKM2 activator groups compared to PGK1 and PKM2 inhibitor groups.
• Adding PGK1 and PKM2 activators promoted desmin degradation, μ-calpain activity and caspase-3 abundance.
• Troponin-T degradation with the higher level in PGK1 activator group than that in PGK1 inhibitor group.
• The degradation of troponin-T was promoted both in PKM2 activator and inhibitor groups.

## 1 Introduction

A series of biochemical and metabolic changes occur during the transformation of the muscle to meat (Chauhan and England, 2018, Huang et al., 2020, Matarneh et al., 2021). Postmortem muscle glycolysis, an essential energy metabolism pathway, directly influences the final meat quality development (Chauhan and England, 2018, Hamm, 1977). The role of adenosine triphosphate (ATP) in regulating postmortem glycolysis has been demonstrated to have a significant effect on the development of postmortem meat quality (Hamm, 1977). After bleeding of animals, ATP is synthesized through the decomposition and metabolism of stored glycogen, ultimately producing lactic acid due to the cessation of oxygen supply (Scheffler & Gerrard, 2007). Consequently, the utilization of ATP results in the production lactate and accumulation of H^+^ ions, which subsequently influences the postmortem muscle pH value. In generally, the degree of pH reduction determines the potential ability of glycolysis, excessive glycolysis leads to the formation of PSE (pale, soft, and exudative) meat or meat with PSE-like characteristics, while inadequate glycolysis results in the formation of DFD (dark, firm, and dry) meat (Briskey, 1964).

In the glycolysis, phosphoglycerate kinase-1 (PGK1), the first enzyme responsible for generating ATP in the process of glycolysis, catalyzes the transformation of 1,3-diphosphoglycerate (1,3-BPG) into 3-phosphoglycerate (3-PG) while simultaneously producing one molecule of ATP (Bernstein & Hol, 1998). Moreover, pyruvate kinase (PK) facilitates the transference of phosphate from phosphoenolpyruvate (PEP) towards ADP, resulting in the production of ATP and the formation of pyruvate in the final step of glycolysis (Israelsen & Vander Heiden, 2015). Mammals have four isoforms of pyruvate kinase (PK): PKL and PKR are presented in the liver, kidney, and erythrocytes, respectively, while PKM1 and PKM2 are presented in various cell types and tissues. Among these isoforms, PKM2 plays an essential role in catalyzing the glycolysis final step, and its activity is subject to regulation by glycolytic intermediates (Yang & Lu, 2015). Many studies have revealed that the different abundance of PGK1 and PKM2 proteins are closely related to meat quality attributes by proteomics approaches (Huang et al., 2023, Huang et al., 2023, Kim et al., 2019). Moreover, Bai et al. (2023) reported that the muscle chilling treatment could influence the PGK activity by regulate its phosphorylation and acetylation level, thus influencing the postmortem meat quality. The higher pyruvate kinase activity with the better meat color, tenderness and WHC, which was found by Ren, Li, Bai, Schroyen, & Zhang. (2022). The study also found that the protein phosphorylation could affect pyruvate kinase activity to regulate postmortem glycolysis (Chen et al., 2019). Furthermore, our previous studies have demonstrated a close association between the activity of PKM2 and PGK1 as well as lamb meat quality traits. They can be used as valuable biological markers for characterizing lamb meat quality (Huang et al., 2023, Huang et al., 2023, Huang et al., 2023, Huang et al., 2023). Therefore, based on current research progress, it has been primarily observed that PGK1 and PKM2 are closely associated with meat quality. However, it is currently uncertain how glycolytic enzymes (PKM2 and PGK1) affect the changes in the development of meat quality traits.

Postmortem muscles attempt to keep cellular energy balance by preserving ATP level within a normal range (Wang, Matarneh, Gerrard, & Tan, 2022). In postmortem muscle, glycolysis is the only predominant pathway for ATP production. Generally, after bleeding of animals, the interruption of oxygen and nutrient supply to all skeletal muscle cells leads to the accumulation of reactive oxygen species (ROS) and gradual depletion of ATP, resulting in the rapid initiation of apoptosis program (Lana & Zolla, 2015). Moreover, the ability of the sarcoplasmic reticulum to accumulate calcium (Ca^2+^) is impaired as a result of pH decline and ATP depletion following slaughter. Consequently, Ca^2+^ is released into the sarcoplasm, leading to the activation of μ-calpain, which impacts meat quality (Bhat, Morton, Mason, & Bekhit, 2018). Skeletal muscle accounts for around 30–40 % of the overall body mass and plays a vital role in protein and energy metabolism. In livestock species, skeletal muscle functions as the primary precursor to meat, and the postmortem maturation of muscle into meat represents a pivotal process that exerts a substantial influence on the overall meat quality. As rigor mortis sets in, the energy reserves of the muscle, including ATP and glycogen, become depleted. This depletion can influence the degradation of muscle proteins and subsequently affect meat quality (Ouali et al., 2006). Overall, the apoptosis, μ-calpain activity and myofibrillar proteins of postmortem muscle are closely related to ATP level. However, PGK1 and PKM2 as the only 2 ATP-generating enzymes in glycolysis, their relationship with postmortem metabolism and physiochemical changes (μ-calpain, myofibrillar protein and apoptosis) remains uncertain.

Therefore, the purpose of present work was to regulate the activity of PGK1 and PKM2 by adding their inhibitors and activators in glycolysis *vitro* model and incubating at 4 °C for 24 h, respectively. Subsequently, we aimed to investigate the effects of PGK1 and PKM2 on glycolysis process, myofibrillar proteins degradation, μ-calpain activity and apoptosis of postmortem muscle. These findings may contribute to an enhanced comprehension of the molecular mechanisms underlying the development of postmortem meat quality. Furthermore, it will provide valuable insights into the interrelationships between physiochemical changes, and metabolic pathways occurring during muscle conversion into meat.

## 2 Materials and methods

### 2.1 Samples collection

Twelve *Longissimus thoracis* (LT) muscle samples were obtained from six to eight-month-old Tan sheep provided by Ningxia Yanchi Tan Sheep Industry Development Group Co., Ltd, China. The sheep were raised under the same feeding regimes: the conventional grazing system (free-range grazing on desertification-prone grasslands in Yanchi County) in conjunction with a supplementary regimen featuring corn. The slaughter of all sheep was conducted in adherence to the cutting technical specification of mutton (NY/T 1564–2007, the Ministry of Agriculture of the People's Republic of China, 2007). And the left LT muscles were removed within 30 min after bleeding. The collected samples were immediately preserved in liquid nitrogen and subsequently stored at −80℃ until further processing.

### 2.2 Experimental design

**Fig. 1:**
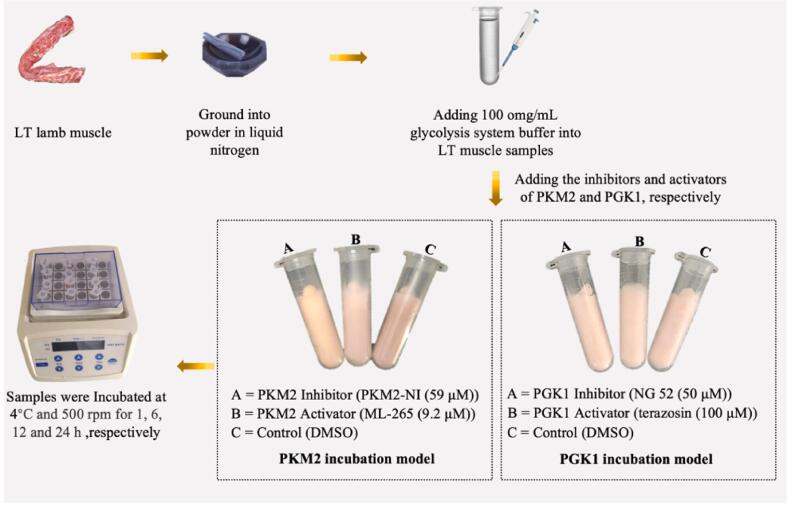
Experimental design for the effects of the activity of the key glycolytic enzymes (PGK1 and PKM2) on the postmortem muscle physiochemical changes.

Construction of the glycolysis *vitro* model referenced the previous studies (Scheffler, Matarneh, England, & Gerrard, 2015). The experimental design presented in Fig. 1. Muscle samples groups were grinded into the powder in the liquid nitrogen, and then added 100 mg/mL glycolysis system buffer (40 mM glycogen, 60 mM KCl, 30 mM creatine, 25 mM carnosine, 5 mM MgCl_2_, 5 mM ATP, 0.5 mM ADP, 10 mM Na_2_HPO_4_, 10 mM sodium acetate, and 0.5 mM NAD^+^, pH = 7.4) and homogenized on ice. Subsequently, the inhibitors of PKM2 (PKM2-IN-1 (59 μM), HY-103617, MedChemExpress, New Jersey, USA) and PGK1 (NG 52 (50 μM), A12494, MedChemExpress, New Jersey, USA), the activators of PKM2 (ML-265 (9.2 μM), HY-182567, MedChemExpress, New Jersey, USA) and PGK1 (terazosin (100 μM), GC13085, Glpbio, Montclair, CA, USA) and control (DMSO) were added into the reaction solution. Following homogenization, the tubes were gently inverted to ensure thorough mixing and subsequently placed in a Thermo Shaker (JXH-100, TUOHE, Shanghai, China) set at 4 °C and 500 rpm. The samples were then collected at 1, 6, 12, and 24 h and preserved at −80 °C for subsequent analysis. In this experiment, a total of 12 LT muscle samples were mixed in pairs of equal weight and divided into 6 groups, and six independent experiments were performed with three technical repeats for each treatment group within the batch.

### 2.3 PKM2 and PGK1 activity

Pyruvate kinase activity and 3-phosphoglycerate kinase activity were determined using the assay kits (BC0545 and BC2255, Solarbio, China) following the instructions provided by the manufacturer. The enzymes activity was presented as units per milligram of protein (U/mg protein).

### 2.4 pH, glycogen, lactate and ATP contents

pH value of the incubation model was measured using a portable pH meter (FE20, Switzerland). The pH meter probe was inserted into the buffer, and three technical replicates of each sample were measured. The levels of glycogen, lactate, and ATP were analyzed using assay kits (BC0345, BC2235, BC0305, Solarbio, China) according to their specifications.

### 2.5 Western blotting analysis

The degradation of desmin, troponin-T, and μ-calpain, and the relative abundance of caspase-3 were analyzed by western blotting approach. The detailed experimental procedure of western blotting was described as our previous study (Huang et al., 2023, Huang et al., 2023). The samples were lysed on ice by RIPA lysis buffer (Sigma, USA) added to protease inhibitor cocktail (Sigma, USA), and then the protein concentration was determined by the BCA protein assay kit (Thermo Fisher Scientific Inc. Waltham, MA). The equal amounts of the protein lysates were performed by 10 %-12 % of separating gel and 4 % of stacking gel of SDS-PAGE and transferred to PVDF blotting membrane (Millipore, Darmstadt, Germany). PVDF membranes were blocked with 5 % nonfat dry milk in TBST solution (137 mM NaCl, 20 mM Tris, 0.1 % Tween-20) for 60 min at room temperature, PVDF membranes were incubated at 4℃ for the night overnight with the following first antibodies: desmin (D1033, Sigma-Aldrich, USA), troponin-T (T6277, Sigma-Aldrich, USA), μ-calpain (MA3–940, Thermo Scientific, USA), caspase-3 (ab4051, 1:1000, Abcam, USA), and GAPDH (AC001, ABclonal, China). The secondary antibodies (goat anti-mouse IgG H&L (HRP) (ab6879, 1:1000, Abcam, USA), and goat anti-rabbit IgG H&L (HRP) (AS014, ABclonal, China)) was used in this study. The protein bands were performed using an ECL detection kit and captured using the ChemiDocTMMP imaging system. The targeted protein bands' intensity was analyzed according to our previous study (Huang et al., 2023, Huang et al., 2023). The muscle sample was collected at 30 min postmortem as a reference sample. Each gel was loaded with a same reference sample. For the degradation of desmin and troponin-T as well as the relative abundance of caspase-3, the targeted bands intensity was quantified by a relative ratio compared with the reference protein of GAPDH band intensity firstly, and then compared with the reference sample band intensity again to calculate it.

### 2.6 Statistical analysis

Data was analyzed by the SPSS Statistics 26.0 (IBM Corp, USA). A General Linear Model analysis was used with treatment group and incubation time and interaction of treatment group × incubation time as fixed effects, number of in vitro experiments (six technical repeats) as random effect. All data were analyzed using one-way analysis of variance (ANOVA), and means were compared using the Duncan’s multiple extreme difference test. *P < 0.05* was considered significant. All results were expressed as the mean ± standard error of the mean (SEM).

## 3 Results

### 3.1 PGK1and PKM2 abilities

**Fig. 2:**
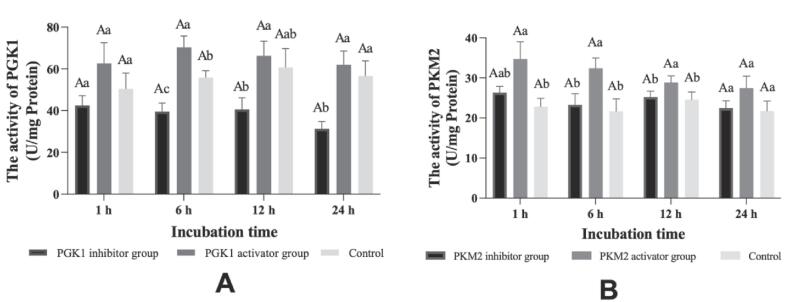
The activity of PGK1 and PKM2 in glycolysis *vitro* system at 1, 6, 12 and 24 h incubation in different treatments. A: PGK1 (phosphoglycerate kinase 1), B: PKM2 (pyruvate kinase 2). A-D: Different letters are significantly different at different incubation time within the same group (*P < 0.05*). a-c: Different letters at the same time points are significantly different between groups (*P < 0.05*).

The results of the addition of activators and inhibitors for PGK1 and PKM2 in postmortem glycolysis *vitro* system to regulate their activity were presented in Fig. 2. During the whole incubation process of the glycolysis *vitro* model, no significant differences were observed in the activity of PGK1 and PKM2 with increasing incubation time (*P > 0.05*). The activator group revealed remarkably higher levels of PGK1 activity than the PGK1 inhibitor group at the 6, 12, and 24 h incubation time points (*P < 0.05,* Fig. 2A). The inhibitor group showed remarkably lower activity of PKM2 compared to the activator group at 6 and 12 h (*P < 0.05*). However, the activity of PKM2 in the activator group showed no remarkable difference compared to the control group (*P > 0.05,* Fig. 2B). In addition, the activity of PGK1 and PKM2 was affected by the inhibitor and activator treatment (*P < 0.001*, Table S1). Therefore, the results suggested that the addition of activators or inhibitors for PGK1 and PKM2 in glycolysis *vitro* model could regulate their activity, respectively.

### 3.2 pH, glycogen, lactate and ATP contents

**Fig. 3:**
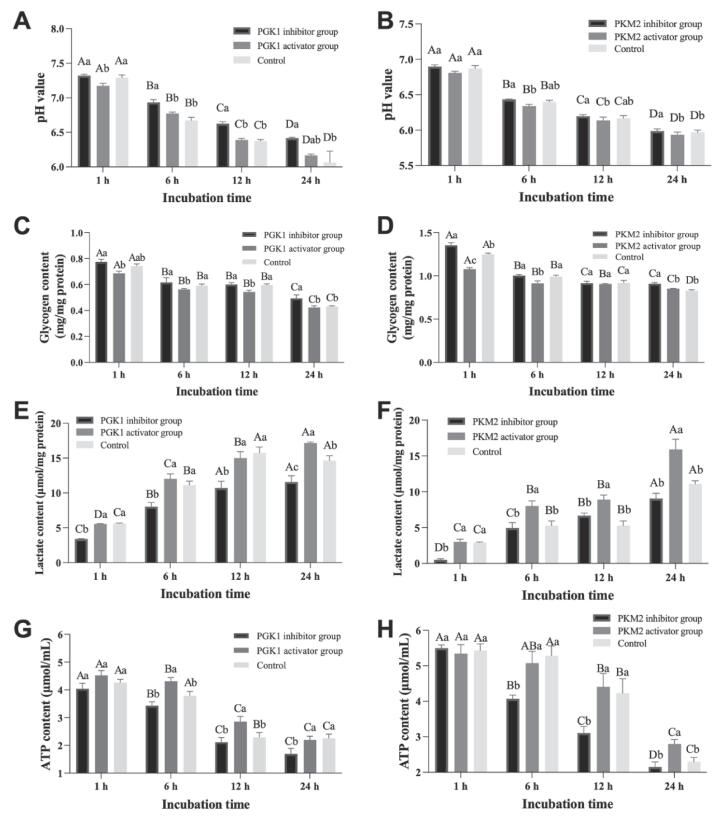
Effect of different treatment groups on pH, glycogen, lactate and ATP contents in glycolysis *vitro* system at 1, 6, 12 and 24 h incubation. A-D: Different letters are significantly different at different incubation time within the same group (*P < 0.05*). a-c: Different letters at the same time points are significantly different between groups (*P < 0.05*).

The addition of activators and inhibitors for PGK1 and PKM2 in postmortem glycolysis *vitro* model to influence the glycolysis process were presented in Fig. 3. In both the PGK1 and PKM2 incubation systems, the pH value showed a significant decrease with increasing of incubation time (*P < 0.05*, Fig. 3A and 3B). Except at the 24 h, the pH value in the PGK1 inhibitor group was remarkably higher than the PGK1 activator group (*P < 0.05*, Fig. 3A). In contrast, the PGK1 activator group showed no significant difference compared to the control (*P > 0.05*). In PKM2 incubation system, the pH value in the PKM2 activator group was significantly lower than that in the PKM2 inhibitor group at the 6, 12, and 24 h (*P < 0.05*, Fig. 3B), and there was no remarkable difference between the PKM2 activator group and the control during the whole incubation time (*P > 0.05*). Moreover, the pH value in postmortem glycolysis *vitro* system was influenced by inhibitor and activator treatment as well as incubation time (*P < 0.05*, Table S1). It was concluded that the addition of activators for PGK1 and PKM2 could promote the decline of pH value. For muscle glycogen content, a decrease in glycogen content was found both in PGK1 and PKM2 treatment groups during the process of incubation (*P < 0.05*, Fig. 3C and 3D). Meanwhile, the glycogen content was affected by inhibitor and activator treatment as well as incubation time (*P < 0.05*, Table S1). In addition, at the 1, 6, and 12 h incubation time points, the glycogen content in the PGK1 activator group was significantly lower compared to both the PGK1 inhibitor group and the control (*P > 0.05*). Throughout the entire incubation time, except for the 24 h time point, there was no remarkable difference observed between the PGK1 inhibitor group and the control (*P > 0.05*). At 1 h, the glycogen content in PKM2 inhibitor group had a remarkably higher than the control (*P < 0.05*), and the glycogen content in control was remarkably higher than PKM2 activator group (*P < 0.05*). The lactate content was influenced by an interaction between treatment and incubation time (*P < 0.05*, Table S1) in postmortem glycolysis *vitro* system. Overall, an increase in lactate level was presented in the PGK1 and PKM2 incubation inhibitors and activators treatment at the entire incubation time (*P < 0.05*, Fig. 3E and 3F). And the lactate content in inhibitor group was significant difference than activator group both in PGK1 and PKM2 incubation (*P < 0.05*), which agreed with the pH value. As presented in Table S1, the ATP level was gradually declined during the process of incubation time, and affected by inhibitor and activator treatment as well as incubation time (*P < 0.05*). Except for 1 h, the ATP content both in PGK1 and PKM2 inhibitor groups was remarkably lower than PGK1 and PKM2 activator groups (*P < 0.05*, Fig. 3G and 3H). The ATP content in PGK1 activator group with a higher level than control at 6 and 12 h (*P < 0.05*), and the ATP content in PKM2 inhibitor group was remarkably lower than control at 6, 12 and 24 h (*P < 0.05*). Therefore, it was concluded that compared to PGK1 and PKM2 inhibitor groups, the addition of PGK1 and PKM2 activators in postmortem glycolysis *vitro* system may accelerate the glycogen consumption, and the production of ATP and lactate, and then the buildup of H^+^ result in the decline of pH value. Therefore, it was suggested that the activity of PGK1 and PKM2 could directly influence and regulate the rate of glycolysis process and then affect the postmortem meat quality.

### 3.3 Myofibrillar proteins degradation

**Fig. 4:**
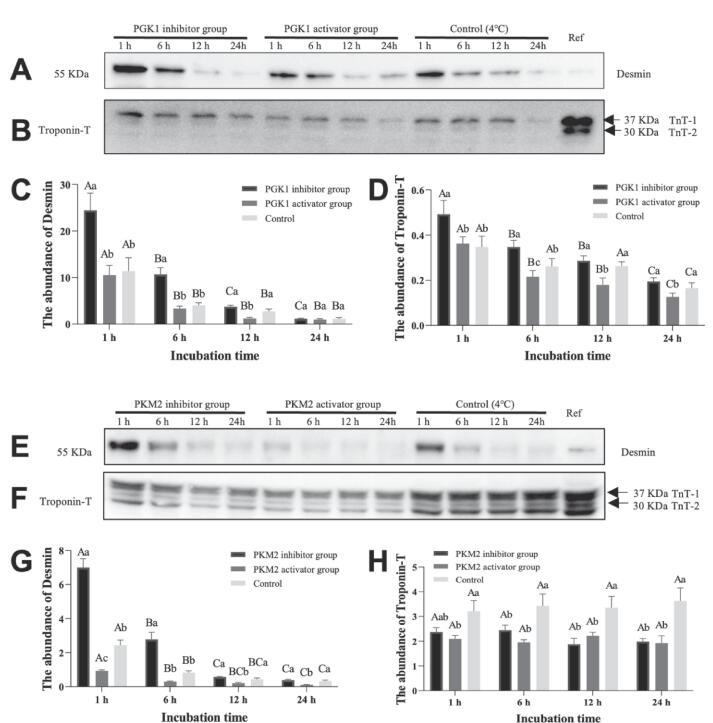
Effect of different treatment groups on the degradation of desmin and troponin in glycolysis *vitro* system at 1, 6, 12 and 24 h incubation. A-D: Different letters are significantly different at different incubation time within the same group (*P < 0.05*). a-c: Different letters at the same time points are significantly different between groups (*P < 0.05*).

Myofibrillar proteins (desmin and troponin-T) degradation in postmortem glycolysis *vitro* system was analyzed by western blot approach and presented in Fig. 4. In PGK1 incubation system, the relative abundance of desmin and troponin-T gradually declined in different groups (*P < 0.05*, Fig. 4A, 4B, 4C and 4D). Desmin relative abundance in PGK1 inhibitor group was significantly higher than PGK1 activator group and control at incubation of 1, 6 and 12 h (*P < 0.05*). There was no remarkable difference between of the PGK1 activator group and control (*P > 0.05*). While the PGK1 inhibitor group with a remarkably higher relative quantity of troponin-T compared to PGK1 activator group at the entire incubation time (*P < 0.05*), and the troponin-T relative abundance in the PGK1 activator group was significantly lower than control (*P < 0.05*). Besides, the relative abundance of desmin was affected by the inhibitor and activator treatment as well as incubation time (*P* < 0.001, Table S1), but the troponin-T relative abundance was only affected by inhibitor and activator treatment (*P* < 0.05). For PKM2 incubation system, the relative abundance of desmin was affected by an interaction between inhibitor and activator treatment as well as incubation time (*P < 0.001*, Table S1). Overall, a decline in desmin relative abundance was observed in three different PKM2 treatment groups during the incubation process (*P < 0.05*, Fig. 4E and 4G). At 1 h, the relative abundance of desmin had a significant variation in different treatment groups (*P* < 0.05), and the PKM2 inhibitor group with the highest, and PKM2 activator group with the lowest. At 6, 12 and 24 h, desmin relative abundance in PKM2 inhibitor group was significantly higher than PKM2 activator group (*P* < 0.05). In contrast, the findings showed that troponin-T relative abundance in control group was remarkably higher than PKM2 inhibitor and activator groups (*P* < 0.05, Fig. 4F and 4H), but there was no significant difference between the PKM2 inhibitor group and PKM2 activator group (*P* > 0.05). Therefore, the results indicated that the addition of PGK1 activator could promote the degradation of desmin and troponin-T compared with the PGK1 inhibitor group. In addition, the addition of PKM2 activator could promote the degradation of desmin compared with the PKM2 inhibitor group and control, but adding the inhibitor and activator for PKM2 could accelerate the degradation of troponin-T.

### 3.4 μ-calpain activity

**Fig. 5:**
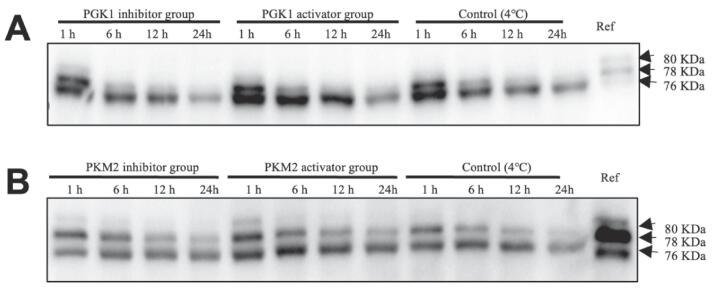
Effect of different treatment groups on the degradation of μ-calpain in glycolysis *vitro* system at 1, 6, 12 and 24 h incubation.

As revealed in Fig. 5, the μ-calpain gradually degraded from 80 kDa to 78 kDa and 76 kDa, both in PGK1 and PKM2 incubation system during the whole incubation process. During the process of incubation, the degradation degree of μ-calpain significantly increased in three different treatment groups in both PGK1 and PKM2 incubation system. Moreover, the 76 kDa value of grey density in PGK1 and PKM2 activator groups was remarkably stronger than that in PGK1 and PKM2 inhibitor groups as well as control, and the PGK1 and PKM2 inhibitor groups with the weaker 76 kDa value of grey density compared to control. Therefore, the results suggested that the addition of PGK1 and PKM2 activators in glycolysis *vitro* system could improve the μ-calpain activity compared to their inhibitor groups, which agreed with the result of desmin degradation.

### 3.5 Caspase-3 relative abundance

**Fig. 6:**
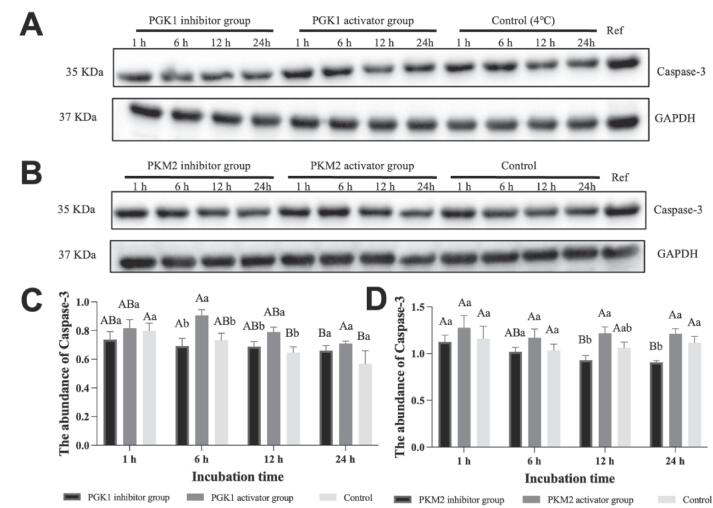
Effect of different treatment groups on the abundance of caspase-3 in glycolysis *vitro* system at 1, 6, 12 and 24 h incubation. A-D: Different letters are significantly different at different incubation time within the same group (*P < 0.05*). a-c: Different letters at the same time points are significantly different between groups (*P < 0.05*).

The abundance level of caspase-3 in PGK1 and PKM2 glycolysis vitro system was performed by western blotting analysis and revealed in Fig. 6. For PGK1 incubation system, the addition of PGK1 activator and inhibitor treatment had a significant difference on caspase-3 relative abundance (*P < 0.001*, Table S1), but the caspase-3 relative abundance was not influenced by incubation time (*P = 0.209*). At the incubation of 6 and 12 h, the abundance level of caspase-3 in PGK1 inhibitor group and control was remarkably lower than the PGK1 activator group (*P < 0.05*, Fig. 6A and 6C), but there was no remarkable variation between PGK1 inhibitor group and control (*P > 0.05*). For PKM2 incubation system, the abundance of caspase-3 in the PKM2 inhibitor group with a remarkably lower level compared to the PKM2 activator group at the incubation of 12 and 24 h (*P < 0.05*, Fig. 6B and 6D). Moreover, the relative abundance of caspase-3 was influenced by the PKM2 activator and inhibitor treatment (*P = 0.003*) as well as incubation time (*P = 0.006*, Table S1). Therefore, it was indicated that the addition of PGK1 and PKM2 activators in glycolysis *vitro* with a higher caspase-3 relative abundance compared to the PGK1 and PKM2 inhibitor groups.

## 4 Discussion

The postmortem glycolysis pathway plays an essential role in the physiological and biochemical metabolism that occurs during the transition of muscle into meat (Hamm, 1977). Following the exsanguination of animals, the nutrient matrix and oxygen supply of muscle cells are interrupted, muscle glycogen is degraded through glycogenolysis and glycolysis pathway to produce the ATP and lactate resulting from anaerobic metabolism, thus leading to pH drops. The previous studies have proved that the fast and inordinate glycolysis results in PSE meat, while inadequate glycolysis leads to DFD meat (Hamm, 1977, Matarneh et al., 2021). PGK1 and PKM2 are the only 2 ATP-generating enzymes that play an important role in postmortem glycolysis pathway. Moreover, PGK1 and PKM2 are also considered as the potential markers to assess and characterize meat quality (Bai et al., 2023, Chen et al., 2019, Huang et al., 2023, Huang et al., 2023, Ren et al., 2022). In this study, the results found that compared to PGK1 and PKM2 inhibitor groups, the addition of PGK1 and PKM2 activators in postmortem glycolysis *vitro* system could accelerate the degradation of muscle glycogen, and the production of ATP and lactate, and then increase the buildup of H^+^ lead to the decline of pH value. The observation revealed that the terazosin could activate PGK1 activity and promote the ATP generation (Chen et al., 2015), this is due to that ATP release is likely to be a key rate-limiting step for the enzymatic reaction (Geerlof, Schmidt, Travers, & Barman, 1997), terazosin may kinetically facilitate the reaction through accelerating ATP release. Nie et al. (2020) reported that the higher PGK1 activity can increase glucose consumption and lactate production, and decrease ADP/ATP ratio. Cai et al. (2019) found that the addition of PGK1 activator could increase levels of glycolysis and cellular ATP. These findings agreed with our results. However, Bai et al. (2023) showed that the postmortem muscle chilling group (25.1 ℃/h) with the higher levels of the glycogen and ATP contents, pH value and PGK1 activity, and lower lactate content than those in control group (4.8 ℃/h). This is maybe due to the fact that the chilling treatment alters metabolic patterns by regulating the levels of phosphorylation and acetylation of glycolytic enzymes, thereby delaying the glycolysis process. In addition, previous studies also have proved that the addition of PKM2 inhibitor could delay the glycolysis by declining the glycogen consumption, as well as ATP and lactate production (Shankar Babu et al., 2018, Sun et al., 2021). Shen et al. (2022) revealed that a significant positive correlation between PKM gene expression and lactate production in pork muscle, while a negative correlation was observed with pH value. Therefore, it was indicated that the higher activity of PGK1 and PKM2 may promote the postmortem glycolytic process and then influenced meat quality.

μ-calpain, an intracellular cysteine protease dependent on calcium, is distributed in various regions of the skeletal muscle sarcomere, predominantly in the Z line. It exists as a heterodimer composed of a catalytic subunit (80 kDa) and a regulatory subunit (28 kDa) (Goll, Thompson, Li, Wei, & Cong, 2003). The 80 kDa subunit of μ-calpain undergoes autolysis, resulting in its conversion to 78 kDa and further to 76 kDa. Activation of μ-calpain occurs in the presence of Ca^2+^, leading to the degradation of the 80 kDa subunit and facilitating postmortem meat tenderization. In this study, we found that the addition of PGK1 and PKM2 activators in postmortem glycolysis *vitro* system could improve the degradation of μ-calpain compared to their inhibitor groups. In general, the postmortem muscle cells come across less energy and more acidic conditions, which can make the sarcoplasmic reticulum dysfunction, and lead to the release of Ca^2+^into the muscle plasma to activate μ-calpain (Warner et al., 2022). Moreover, previous study also found that the rapid decline of postmortem pH value in the initial stages plays a crucial role in the autolysis and activation of μ-calpain. A faster pH decline is considered advantageous as it facilitates accelerated proteolysis of known calpain substrates (Bhat et al., 2018). Rhee & Kim. (2001) reported that the low voltage electrical stimulation combined with temperature (30℃ treatment) for early postmortem beef could increase the glycolytic rates and improve the μ/m-calpain activity. These results were consistent with our study. Thus, it was suggested that the addition of PGK1 and PKM2 activators could improve μ-calpain activity in postmortem muscle.

Desmin and troponin-T are structural proteins found in muscle tissue, and they play important roles in muscle contraction. Desmin, a crucial intermediate filament protein, is found in all muscle cells and plays a vital role in linking myofibrils to Z-disks, as well as connecting them to sarcolemma and mitochondria (Ali et al., 2022). In this study, compared to the PGK1 and PKM2 inhibitor groups, desmin degradation had a remarkably higher level in PGK1 and PKM2 activator groups, which agreed with the result of μ-calpain activity. It was mainly because of the increase of μ-calpain activity in PGK1 and PKM2 activator groups promoted the proteolysis of desmin, which led to the increase of desmin degradation (Koohmaraie, 1992). Moreover, our results found that troponin-T degradation had a remarkably higher level in PGK1 activator group than that in PGK1 inhibitor group, which was similar with the result of desmin degradation. The reason was that troponin-T was considered as a reflective substrate of μ-calpain can be degraded to generate a 30 kDa degradation product (Li, Li, Gao, Du, & Zhang, 2017). Interestingly, the degradation of troponin-T in PKM2 inhibitor and activator groups was remarkably higher than control, while there was no significant different between the PKM2 inhibitor and activator groups. This was indicated that the addition of PKM2 inhibitor and activator in postmortem glycolysis *vitro* system could promote the degradation of troponin-T. Actually, the level of troponin-T degradation by endogenous enzymes is considered as the key indicator to reflect the postmortem meat tenderness (Ouali, 1990). According to a previous study, it was reported that oxidation has an impact on the degradation of myosin heavy chain and α-actinin, increasing their degradation by μ-calpain. However, the degradation of troponin-T by μ-calpain is reduced in the presence of oxidation. This discrepancy is attributed to the oxidative modification of troponin-T, which alters its susceptibility to μ-calpain (Xue, Huang, Huang, & Zhou, 2012). Moreover, Ding, Wei, Zhang, Zhang, & Huang. (2021) found that troponin-T degradation was delayed by oxidation, while the degradation of desmin was accelerated. This difference could be attributed to the oxidative modification of myofibrils, which led to the different susceptibility to proteolysis. Therefore, we inferred that the addition of PKM2 inhibitor and activator may increase the sensitivity of troponin-T to hydrolysis, thereby promoting troponin-T degradation.

After the bleeding of animals, the nutrient matrix and oxygen supply of muscle cells are interrupted, resulting in severe stress changes such as massive accumulation of ROS, gradual depletion of ATP, which will inevitably lead to apoptosis (Lana et al., 2015). At the beginning of apoptosis process, the cytochrome *c* (Cyt-c) is released from the cytoplasm. Cyt-c is then combined with apoptotic protease activating factors 1 (Apaf-1) to form apoptotic bodies that further activate caspase-9, and the activated caspase-9 further activates caspase-3, which results in apoptosis cascade. Caspase-3 is widely considered a critical terminal protease that participates in the apoptosis pathway (Kemp & Parr, 2008). In this study, the results revealed a significant increase in the relative abundance of caspase-3 in the PGK1 and PKM2 activator groups compared to the PGK1 and PKM2 inhibitor groups (*P < 0.05*). The previous study reported that in the presence of abundant high-energy phosphate-rich metabolites, such as ATP, protein phosphorylation could occur concurrently or play a role in the apoptotic cascade reaction (D'Alessandro & Zolla, 2013). It was indicated that the addition of the PGK1 and PKM2 activators could promote the glycolysis, increase ATP production and subsequently lead to the apoptosis. In addition, the previous findings demonstrated that caspase-3 can make the degradation of the key myofibrillar proteins once the release of cytochrome *c* and activation of caspase-3 occurred in the early-stage postmortem (Ding et al., 2021, Kemp and Parr, 2008, Lana and Zolla, 2015). Therefore, the addition of PGK1 and PKM2 activators could improve the relative abundance of caspase-3 and then promote the degradation of myofibrillar proteins, which agreed with the result of desmin degradation.

**Fig. 7:**
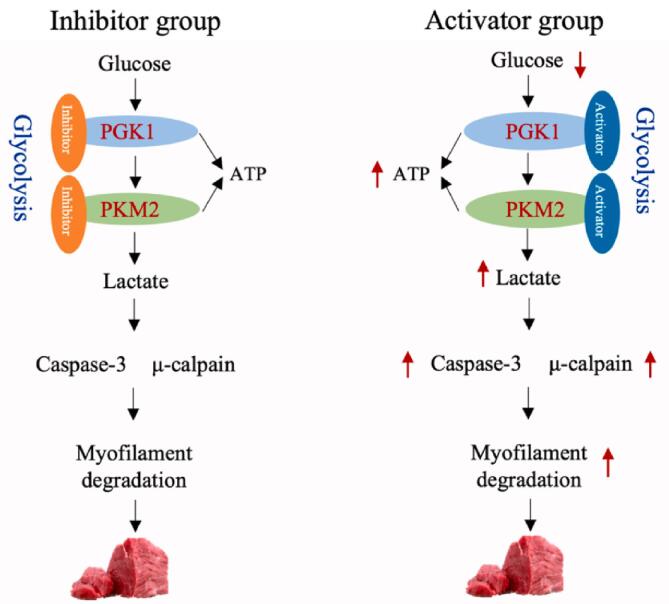
Possible mechanisms of the effects of PGK1 and PKM2 on postmortem muscle metabolism and physicochemical changes.

In summary, as the exclusive ATP-generating enzymes in glycolysis, PGK1 and PKM2 play a central role in the interconnected processes of glycolysis, apoptosis, μ-calpain activity, and the degradation of myofibrillar proteins in postmortem muscle, all of which are intricately linked to ATP levels. Consequently, the modulation of PGK1 and PKM2 is likely to influence postmortem glycolysis, μ-calpain activity, myofibrillar protein degradation, and apoptosis, thereby impacting postmortem meat quality through the regulation of ATP levels. Therefore, compared to inhibitor groups, the ATP and lactate contents in the PGK1 and PKM2 activator groups remarkably increased, while the glycogen content decreased. Moreover, the addition of PGK1 and PKM2 activators could improve the degradation of desmin, the activity of μ-calpain, and the relative abundance of caspase-3 (Fig. 7).

## 5 Conclusion

The present work investigated the influence of PGK1 and PKM2 on the postmortem muscle metabolism and physiochemical changes. The results indicated that the addition of PGK1 and PKM2 activator could increase the ATP and lactate levels and glycogen consumption. Moreover, incorporating PGK1 and PKM2 activators could improve desmin degradation, μ-calpain activity, and caspase-3 relative abundance. However, the degradation of troponin-T was promoted in PGK1 activator group compared with PGK1 inhibitor group, while the degradation of troponin-T was promoted in both PKM2 inhibitor and activator groups. This was maybe due to the addition of PKM2 inhibitor and activator may increase the sensitivity of troponin-T to hydrolysis, thereby promoting the degradation of troponin-T. Overall, it was concluded that the activity of PGK1 and PKM2 could affect glycolysis process, myofibrillar proteins, μ-calpain and apoptosis pathways and further affect the development of postmortem meat quality. Furthermore, the future research could study constructing meat quality prediction models based on PGK1 and PKM2 and developing non-destructive online detection technology for meat quality assessment.

## CRediT authorship contribution statement

**Caiyan Huang:** Conceptualization, Methodology, Software, Writing – original draft, Writing – review & editing. **Dequan Zhang:** Investigation, Supervision, Validation. **Christophe Blecker:** Conceptualization, Supervision. **Yingxin Zhao:** Data curation, Resources. **Can Xiang:** Formal analysis, Methodology. **Zhenyu Wang:** Investigation, Supervision. **Shaobo Li:** Investigation. **Li Chen:** Investigation, Supervision, Validation.

## Declaration of competing interest

The authors declare that they have no known competing financial interests or personal relationships that could have appeared to influence the work reported in this paper.

## Data Availability

Data will be made available on request.
