# Supplementary material for: Effects of phosphoglycerate kinase 1 and pyruvate kinase M2 on metabolism and physiochemical changes in postmortem muscle
Source: Food Chem X. 2024 Jan 9;21:101125. doi: 10.1016/j.fochx.2024.101125 (PMC10827398; doi:10.1016/j.fochx.2024.101125)
Supplement: Supplementary data 1 [file mmc1.docx]

**Table S1** Effect of treatment group and incubation time factors as well as their interaction on physicochemical properties of postmortem glycolysis *vitro* system by a general linear model.

|  | Parameters | *P value* | | |
| --- | --- | --- | --- | --- |
|  |  | Treatment | Incubation time | Treatment*Incubation time |
| PGK1 incubation | PGK1 activity | <0.001 | 0.644 | 0.847 |
|  | pH value | <0.001 | <0.001 | 0.123 |
|  | Glycogen content | <0.001 | <0.001 | 0.643 |
|  | Lactic acid content | <0.001 | <0.001 | 0.054 |
|  | ATP content | <0.001 | <0.001 | 0.4468 |
|  | Desmin abundance | <0.001 | <0.001 | <0.001 |
|  | Troponin-T abundance | 0.05 | 0.18 | 0.595 |
|  | Caspase-3 abundance | 0.001 | 0.209 | 0.859 |
| PKM2 incubation | PKM2 activity | <0.001 | 0.349 | 0.651 |
|  | pH value | 0.04 | <0.001 | 0.99 |
|  | Glycogen content | <0.001 | <0.001 | 0.001 |
|  | Lactic acid content | <0.001 | <0.001 | 0.001 |
|  | ATP content | <0.001 | <0.001 | 0.023 |
|  | Desmin abundance | <0.001 | <0.001 | <0.001 |
|  | Troponin-T abundance | <0.001 | 0.965 | 0.741 |
|  | Caspase-3 abundance | 0.003 | 0.006 | 0.502 |
